# The expression of myeloperoxidase in thrombi is associated with reduced heme oxygenase‐1 induction and worse left ventricular remodeling in patients with acute ST‐elevation myocardial infarction

**DOI:** 10.1002/clc.23542

**Published:** 2021-01-06

**Authors:** Xibao Shi, Tianqi Zhu, Jun Ni, Ruiyan Zhang

**Affiliations:** ^1^ Department of Cardiology, Shanghai Ruijin Hospital Shanghai Jiaotong University School of Medicine Shanghai China

**Keywords:** acute myocardial infarction, heme oxygenase‐1, myeloperoxidase, prognosis

## Abstract

**Background:**

Myeloperoxidase (MPO) secreted by neutrophils is the enzyme that kills bacteria and other pathogens. Acute myocardial infarction (AMI) is usually caused by thrombosis in response to vulnerable plaque rupture. Circulating MPO was found to be associated with increased mortality in AMI patients. However, the relationship between MPO in thrombi and the prognosis of AMI patients remains unknown.

**Hypothesis:**

MPO expression in thrombi is associated with the prognosis of patients who underwent primary percutaneous coronary intervention (PCI) after AMI.

**Methods:**

This study included 41 consecutive patients with acute ST‐elevation myocardial infarction, who successfully underwent primary PCI, during which we collected thrombi remaining in the culprit artery using aspiration catheters. These thrombus samples were fixed, and immunohistochemical staining against MPO and heme oxygenase‐1 (HO‐1) was conducted. Enrolled patients were divided into two groups based on the induction of thrombotic MPO, which was quantified using Image J software.

**Methods:**

We observed that increased MPO was associated with lower left ventricular ejection fraction (LVEF) and worse LV remodeling in AMI patients. Instead, patients with decreased thrombotic MPO induction had a considerable improvement in LVEF 1 month after discharge (54.4 ± 2.0% vs. 61.1 ± 2.3%, *p* < 0.01). In the MPO group, a reduction in the thrombotic HO‐1 level contributed to the development of adverse LV remodeling. Logistic regression showed that MPO was a considerable risk factor for adverse LV remodeling (adjusted OR 3.70, *p* < 0.05).

**Conclusion:**

MPO expression in thrombi is associated with reduced LVEF and deteriorated LV remodeling in AMI patients, which may be due to HO‐1 suppression in thrombi.

## 1 INTRODUCTION

Acute ST‐elevation myocardial infarction (STEMI) remains one of the leading causes of cardiovascular death worldwide. Although the trends of cardiac mortality in developed countries have dropped in recent years, AMI is still considered the second killer in adults in China. To date, we already know that the pathological nature of AMI is caused by thrombi that block the coronary arteries. Subsequent ischaemia and hypoxia in the heart lead to symptomatic chest pain and cardiac malfunction if revascularisation is not applied. A previous study revealed that a high thrombus burden in culprit arteries leads to more complications and worse outcomes.^1^ Patients with high thrombus burden are likely to have more major cardiac events and in‐stent restenosis after primary stenting.^2^

Myeloperoxidase (MPO) secreted by inflammatory cells, such as neutrophils, is an enzyme that kills bacteria and other pathogens in the body. It is produced in the granules of neutrophils and is a heme‐binding protein, which acts as a subfamily of peroxidases and produces hypochlorous acid (HOCl) from hydrogen peroxide (H_2_O_2_) and chloride anion (Cl^−^) during the neutrophil respiratory burst.^3^ HOCl is cytotoxic and kills bacteria and other pathogens when infections occur. Rashid et al. demonstrated that MPO is a potential molecular imaging and therapeutic target for the detection of high‐risk atherosclerotic plaque in mice.^4^ A study also reported that circulating MPO is associated with an increase in mortality in patients with AMI,^5^ possibly, because the HOCl produced by MPO could promote endothelial dysfunction, apoptosis, and vascular thrombosis.^6^ In addition, MPO locally contributes to thrombogenic circumstances by enhancing endothelial cells, which release tissue factors, acting as the initial factor of the coagulation cascade. However, when thrombus forms, neutrophils are locked up with activated platelets, red blood cells, and fibrin. Thus, the function of MPO in thrombus, when AMI occurs, remains unclear. Herein, we hypothesize that the expression of MPO in thrombi is associated with prognosis in patients who underwent primary percutaneous coronary intervention (PCI) after AMI.

## 2 MATERIALS AND METHODS

### 2.1 Subjects

This study enrolled 41 patients. In the emergency department, all patients were confirmed to have acute STEMI by symptomatology, electrocardiography, and laboratory tests according to the guidelines of the Joint Task Force of the European society of Cardiology.^7^

All procedures were performed based on the relevant ethical guidelines for human research, were in agreement with the Helsinki Declaration, and were approved by the Ethics Committee of Shanghai Ruijin Hospital. All subjects signed their informed consent to participate in this study.

On admission, all subjects underwent medical examinations and completed a questionnaire on their personal and medical issues, including age, medical history, and medication use. Subjects were excluded from this study if they had cancer, renal failure, liver disease, or inflammatory and/or autoimmune disease. Family history of cardiovascular disease, hypertension (systolic blood pressure > 130 mmHg or diastolic blood pressure > 90 mmHg or taking antihypertensive drug), diabetes (fasting glucose plasma concentrations higher than 7.0 mM or 11.0 mM after 2 h of oral glucose intake or diabetic drug treatment), and dyslipidemia (plasma low‐density lipoprotein cholesterol concentrations >4.3 mM).

### 2.2 Primary PCI and aspiration thrombectomy

Patients underwent primary PCI if they had acute STEMI and ischemic symptoms of <12 h, had acute STEMI and ischemic symptoms <12 h, had contraindications to fibrinolytic therapy, or had STEMI and cardiogenic shock. Blood samples were collected before the primary PCI procedure, and circulating MPO levels were detected using an ELISA kit (Abcam, ab119605). During PCI, coronary angiography revealed that all enrolled subjects had a high thrombus burden in the culprit arteries. Aspiration thrombectomy was performed as before^8^ with a Thrombuster II aspiration catheter (Kaneka, Osaka, Japan) or Export AP aspiration catheter (Medtronic, Min­neapo­lis, MN). The aspirated thrombi were carefully flushed with saline, fixed in 10% (v/v) paraformaldehyde, and finally embedded in paraffin. Cross‐sections (5 μm) were transferred onto slides and stained using rabbit polyclonal anti‐MPO antibody (Abcam, ab9535; dilution 1: 200, Cambridge, UK) and anti‐heme oxygenase‐1 (HO‐1) antibody (Stressgen SPA‐895; dilution 1:100, San Diego, CA). Finally, avidin‐biotin‐horseradish peroxidase was used for detection (Vectorstain Elite ABC kit; Vector Laboratories, Burlingame, CA). As negative controls, the rabbit serum was used as a primary antibody. An upright light microscope (Olympus, Tokyo, Japan) was used to capture images, and positive MPO and HO‐1‐stained areas were carefully quantified and labeled using the Image J software. The ratio of the positively stained area to the whole thrombus area was calculated. In this study, for MPO staining, we considered the ratio of the positively stained area and the whole thrombus area ≥ 50% as MPO+, otherwise as MPO‐.

### 2.3 Statistical analysis

Statistical analysis was performed using GraphPad Prism 8.0. Individual means of different groups were compared by one‐way analysis of variance, followed by post hoc Newman–Keuls tests. To take uneven variances into account, for comparisons between groups of unequal numbers, Welch's correction for Student's t‐test was used. Paired or unpaired t‐tests and chi‐square tests were used as appropriate. A value of *p* < 0.05 was considered statistically significant. Finally, significant variables were entered into a logistic regression equation to test factors that predict LV remodeling (LVEF improvement ≥10%).

### 2.4 Follow‐up after primary PCI

All enrolled patients took prescribed standard medications (including dual antiplatelet, angiotensin‐converting‐enzyme inhibitors, and beta‐blockers) after discharge. We performed follow‐up echocardiography (echo) at 1 month and 3 months to evaluate left ventricular (LV) remodeling. The parameters measured included LV ejection fraction (LVEF), LV diastolic end diameter (LVDED), LV systolic end diameter (LVSED), LV diastolic end volume (LVDEV), and LV systolic end volume (LVSED).

## 3 RESULTS

We successfully conducted MPO and HO‐1 immunochemical staining and found that all subjects were positive in MPO and HO‐1 staining (Figure S1). The enrolled patients were divided into two groups (MPO‐ and MPO+) based on their induction of MPO in thrombi. The clinical manifestations of the two groups are shown in Table 1. No differences were found in age, hypertension, diabetes, and dyslipidemia between the two groups. Even before the primary PCI, the levels of circulating CK‐MB were similar, and a reduction in CK‐MB level was observed in the MPO‐ group (207.1 ± 45.6 vs. 280.8 ± 45.1, *p* < 0.05). On the contrary, the CK‐MB level was increased in the MPO+ group (315.1 ± 48.6 vs. 212.5 ± 41.2, *p* < 0.05). Although not significant, patients in the MPO‐ group were likely to have lower circulating MPO level (709.3 ± 99.7 vs. 1065.0 ± 349.3) and N‐terminal brain‐type natriuretic peptide (NT‐pro‐BNP; 1016.4 ± 249.8 vs. 2193.0 ± 582.1) and higher incidence of single‐vessel diseases (52.6% vs. 36.4%) than their counterparts. Finally, in‐hospital echo test was performed in both groups. The results showed no differences in LVEF, LVDED, LVSED, LVDEV, or LVSED between the two groups (Table 1). Two deaths were recorded (one patient died from cardiogenic shock before discharge; the other died from major cardiovascular events at 2 months after discharge).

**TABLE 1:** Clinical manifestations of enrolled patients

	MPO‐	MPO+	*p* value
Patient number	19	22	n/a
Male	15 (78.9%)	19(86.3%)	0.84
Age (year)	59.8 **±** 2.4	62.8 **±** 2.2	0.38
Hypertension	16 (84.2%)	18 (81.8%)	0.84
Diabetes	16 (84.2%)	17 (77.2%)	0.57
Dyslipidemia	14 (73.7%)	16 (72.7%)	0.94
CK‐MB pre PCI (mg/dl)	280.8 **±** 45.1	212.5 **±** 41.2	0.26
CK‐MB post PCI (mg/dl)	207.1 **±** 45.6	315.1 **±** 48.6	0.31
Circulating MPO (pg/ml)	709.3 ± 99.7	1065.0 ± 349.3	0.35
NT‐pro‐BNP (ng/ml)	1016.4 ± 249.8	2193.0 ± 582.1	0.06
Single‐vessel Disease	10 (52.6%)	8 (36.4%)	0.35
Ischemic time (h)	4.9 ± 1.6	6.3 ± 1.7	0.01
LVEF (%)	54.4 ± 2.0	53.7 **±** 2.3	0.98
LVDED (mm)	52.1 **±** 1.2	51.3 **±** 1.3	0.66
LVSED (mm)	36.8 **±** 1.2	36.2 **±** 1.4	0.73
LVDEV (ml)	126.9 **±** 5.4	129.9 **±** 7.3	0.75
LVSEV (ml)	58.1 **±** 4.0	61.0 **±** 6.5	0.71

*Note:* A *p* < 0.05 is considered as significant.

**FIGURE 1:**
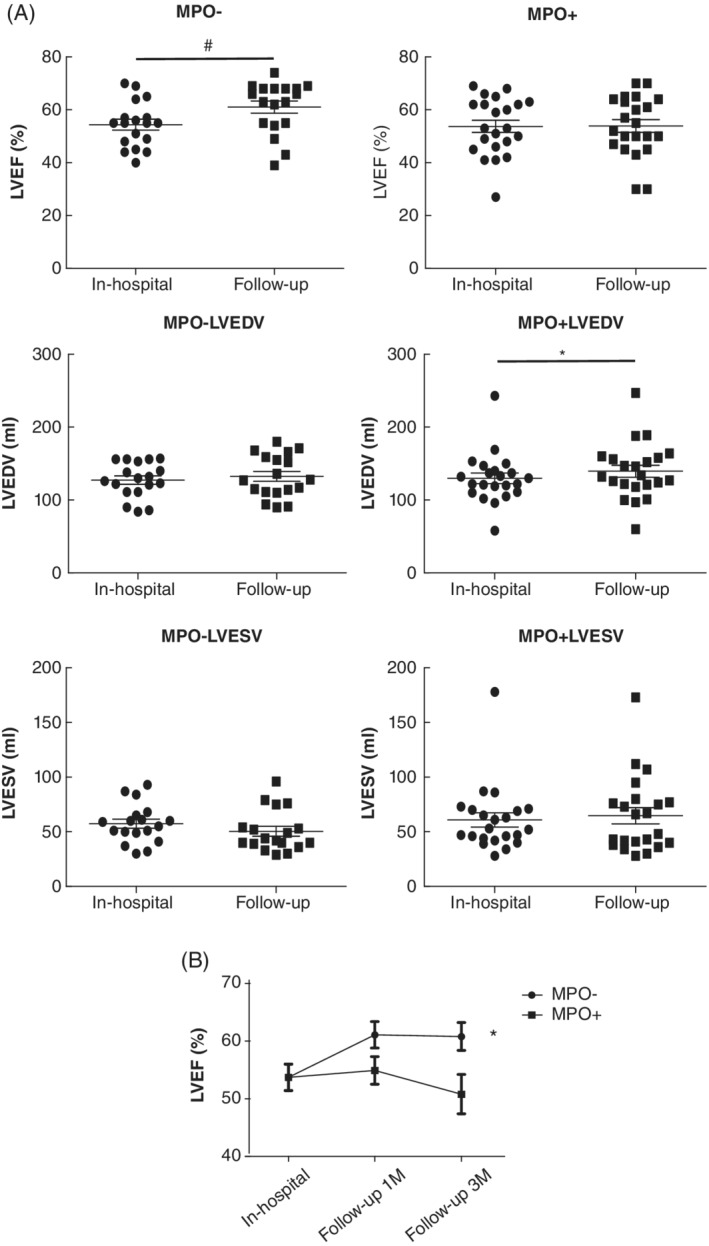
MPO in thrombi is associated with a worse left ventricular remodeling in patients with AMI. (A) LV remodeling between the MPO+ and the MPO‐ groups at 1‐month follow‐up. All enrolled patients underwent echo test at 1‐month follow‐up, and findings were compared with in‐hospital results. Changes in LVEF, LVESV, and LVEDV are displayed in the figure (left panel, MPO‐; right panel, MPO+). Interestingly, in the MPO‐ group, we found a significant increase in LVEF 1 month after discharge (54.4 ± 2.0% vs. 61.1 ± 2.3%, #*p* < 0.01). This improvement in LVEF was caused by a slight increase in LVDEV and a decrease in LVESD. Data are displayed as mean ± SEM. (B) Follow‐up results of LVEF changes after primary PCI in patients with AMI. LVEF tested by echo showed that the MPO‐ group had better LVEF improvement than the MPO+ group at 1‐month and 3‐month follow‐up (* *p* < 0.05). Data are displayed as mean ± SEM. HO‐1, heme oxygenase‐1; LVDEV, left ventricular diastolic end volume; LVEF, left ventricular ejection fraction; LVESD, left ventricular systolic end volume; MPO, myeloperoxidase

During follow‐up, we performed 100% echo test at 1 month and 85% echo test at 3 months in all enrolled patients (Table 2). A significant difference was noted in the LVEF at 1‐month follow‐up (61.1 ± 2.3 vs. 53.9 ± 2.4) between the two groups. However, other echo parameters, including LVDED, LVSED, LVDEV, and LVSED, remained not significantly different between the two groups. Moreover, to reveal LV remodeling after MI in these patients, we performed a statistical analysis of these cardiac parameters in each group (Figure 1(A)). Interestingly, in the MPO‐ group, we found a significant increase in LVEF 1 month after discharge (61.1 ± 2.3 vs. 54.4 ± 2.0, *p* < 0.01). This improvement in LVEF was caused by a slight increase in LVDEV and a decrease in LVESD. Nevertheless, in the MPO+ group, LVEF remained unchanged 1 month after the procedure and decreased at 3 months of follow‐up (Figure 1(B)), which may be due to considerable increases in LVEDV and LVESD, indicating LV enlargement and dysfunction.

**TABLE 2:** Echo parameters at 1 month follow‐up

	MPO‐	MPO+	*p* value
Patient number	18	22	n/a
LVEF (%)	61.1 ± 2.3	53.9 ± 2.4	<0.05
LVDED (mm)	53.1 ± 1.1	52.6 ± 1.4	0.82
LVSED (mm)	36.5 ± 1.3	37.5 ± 1.5	0.16
LVDEV (ml)	130.9 **±** 6.9	139.5 **±** 8.2	0.52
LVSEV (ml)	50.2 **±** 19.6	64.7 **±** 34.7	0.12

*Note:* A *p* < 0.05 is considered as significant.

**FIGURE 2:**
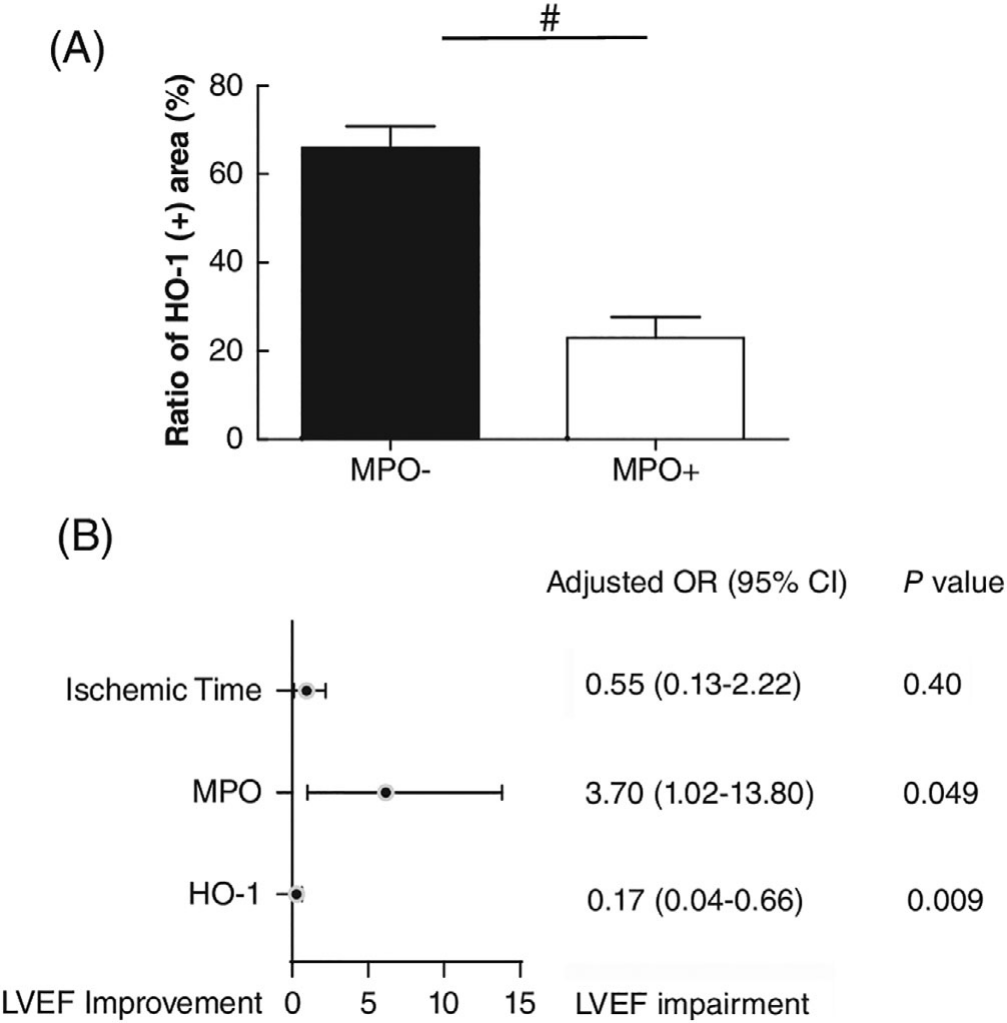
MPO in thrombi attenuated HO‐1 induction and acted as a risk factor predicting LV remodeling after STEMI. (A) Immunochemical staining for HO‐1 was conducted in all thrombotic samples. Positively stained areas were carefully quantified and labeled using Image J software. The ratio of positive HO‐1‐stained area and the whole thrombus area was calculated and compared between the MPO+ group and the MPO‐ group. The result revealed that the ratio of positive HO‐1‐stained area was higher in the MPO‐ group than in the MPO+ group (66.1 ± 4.8 vs. 23.0 ± 4.76, # *p* < 0.01). Data are displayed as mean ± SEM. (B) Three variables including ischemic time, MPO, and HO‐1 were put into a logistic regression equation after determining a 10% increase in LVEF as LVEF improvement. The results showed that MPO was a risk factor for LVEF improvement (adjusted odds ratio 3.70, *p* = 0.49). Nevertheless, HO‐1 (adjusted OR 0.17, *p* = 0.009) was significantly associated with LVEF improvement, and the increase in HO‐1 induction in the thrombus was related to a better prognosis. HO‐1, heme oxygenase‐1; LVEF, left ventricular ejection fraction; MPO, myeloperoxidase; OR, odds ratio; STEMI, ST‐elevation myocardial infarction

Immunostaining revealed a higher level of HO‐1 in thrombi collected from the MPO‐ group than from the MPO+ group (Figure 2(A)). Furthermore, we measured the levels of circulating total bilirubin, which is a product of heme degradation catalyzed by HO‐1. However, we did not find any differences in terms of the levels of circulating bilirubin between the two groups (MPO+ 14.7 ± 2.1 vs MPO‐ 17.6 ± 1.6, *p* > 0.05). Finally, we entered three parameters, including ischemic time, MPO, and HO‐1, into a logistic regression equation after determining a 10% increase in LVEF as a cardiac improvement. We found that MPO (adjusted odds ratio [OR] 3.70, *p* = 0.049) and HO‐1 (adjusted OR 0.17, *p* = 0.009) were significantly associated with LVEF improvement, defined as a 10% increase in LVEF (Figure 2(B)). However, ischemic time (adjusted OR 0.55, *p* = 0.40) failed to achieve significance.

## 4 DISCUSSION

To date, we already know that atherosclerosis is an inflammatory disease. Inflammatory cells, such as neutrophils, secrete enzymes that degrade the fibrous cap on top of the atherosclerosis plaque and subsequently make the plaque vulnerable and then ruptured.^9^ Although MPO is secreted locally by neutrophils, the levels of MPO in the circulation have been shown to be associated with acute STEMI. Goldmann et al. reported that MPO levels were elevated in patients with AMI even at 2 h before the occurrence of myocardial injury.^10^ Further studies have found that MPO concentration is correlated with the severity of coronary stenosis in patients with coronary heart diseases.^11^ Patients with severe lesions in the coronary arteries likely have higher levels of circulating MPO. Other studies have suggested that MPO is a risk factor for predicting mortality after acute coronary syndrome.^12^ However, none of these studies have shown the presence of MPO in thrombus that blocks culprit vessels in AMI. As neutrophils or macrophages secrete MPO locally, we strongly believe that further investigation on thrombotic MPO could help us unveil insights into its function in thrombus. Interestingly, the amount of thrombotic MPO could, or at least partially, reflect the levels of inflammation in or close to the ruptured plaque that caused AMI. The severity of inflammation is an indication of subsequent LV remodeling that predicts prognosis after primary PCI^13^. In this study, patients in the MPO+ group had increased circulating CK‐MB, NT‐pro‐BNP, and MPO and prolonged ischemic time. Furthermore, patients in the MPO+ group were likely to have multivessel diseases. These findings suggest that patients in the MPO+ group had high levels of oxidative stress and severe inflammation, causing further myocardial damage/necrosis and therefore being associated with a reduction of cardiac functions found during follow‐up.

LV remodeling is commonly observed in patients with AMI.^14^ Parameters that evaluate LV remodeling by echo are powerful predictors of prognosis for these patients. In these parameters, LVESV was reported as the major determinant of survival after recovery from myocardial infarction.^15^ LV adverse remodeling, normally determined as an increase in LVEDV, is the main cause of heart failure after an AMI with ST‐segment elevation. Reindl et al. revealed that following primary PCI, an increase of 10% of LVEDV appeared to be associated with the occurrence of major adverse cardiovascular events, including all‐cause death, re‐infarction, stroke, and new‐onset congestive heart failure.^14^ Inflammation is a key component of tissue healing. Clinicians measured inflammatory markers, for example, interleukin (IL)‐1β, before PCI, 2 days after PCI, and 7 days after PCI and found that IL‐1β levels predicted LVESV index and LVEDV index tested by cardiovascular magnetic resonance at 1 year.^16^ In our cohort, due to the small sample size, we did not observe a significant incidence in major adverse cardiovascular events between the two groups during the follow‐up period. Nevertheless, in the MPO+ group, patients had increased LVEDV and decreased LVEF, which was consistent with previous findings. This evidence suggests that even if primary PCI was performed in both groups, the inflammatory levels indicated by thrombotic MPO played an important role in affecting the development of adverse LV remodeling. As a result, further understanding of the mechanistic insights of MPO in AMI appears to be urgent.

HO‐1 is one of the main protein enzymes that degrade heme in the body.^17^ A previous pathological study revealed increased HO‐1 levels in atherosclerotic plaques. In a clinical study, Novo et al. showed that increased HO‐1 levels led to reduced severity of coronary artery diseases in patients with STEMI.^18^ As such, HO‐1 is considered as a protective enzyme against oxidative stress that causes cardiovascular diseases.^19^ Additionally, aged and ovariectomised animals exhibited a considerable increase in MPO activity, a decrease in HO‐1 activity, and significant ST‐segment depression displayed by electrocardiogram.^20^ Our previous findings showed that the expression of HO‐1 in thrombi was associated with LVEF improvement in patients with AMI (data unpublished). A reduced HO‐1 level predicted worse cardiac functions and increased HO‐1 provided protection against cardiac injury. Similarly, in this study, we conducted HO‐1 staining and found that HO‐1 induction in these MPO+ patients was decreased, which could explain our observation that MPO‐ patients had better improvements in LV function and remodeling. The results of the logistic regression also supported the finding that MPO was a considerable risk factor for adverse LV remodeling; on the contrary, an increase in HO‐1 induction in thrombus was related to a better prognosis. A previous animal study demonstrated that HO‐1 induction reduced apoptosis that was caused by hypoxia‐reoxygenation injury and increased proliferation and repair of cardiomyocytes in the infarct border area at a very early stage after infarction.^21^ HO‐1 abrogated both proliferation and apoptosis of cardiomyocytes and attenuated scar formation, thereby alleviating potential adverse LV remodeling. Instead, in this study, the HO‐1 reduction warranted increases in oxidative stress and caused further ischaemia/reperfusion injury of cardiomyocytes. Moreover, our data revealed that the levels of circulating total bilirubin appeared similar in both groups, suggesting that the protective roles played by HO‐1 only remained locally at the origin of the thrombus. This interestingly indicated that a very small amount of HO‐1 secreted locally inhibited LV enlargement and improved LVEF, and these benefits were able to last 3 months.

## 5 STUDY LIMITATIONS

In this study, the number of enrolled patients was very limited. This is because, according to the European society of Cardiology Guidelines for the management of AMI, thrombotic aspiration is only recommended for patients with high thrombus burden. In our hospital, we did not conduct thrombectomy in every patient who underwent primary PCI, and we therefore strongly considered this work as a pilot study. However, before starting this study, we had carefully performed statistical power calculations using average values and found that the current number of enrolled patients had a 100% statistical power. Further clinical investigation with a larger sample size must provide solid evidence in the future. In addition, follow‐up was completed at 3 months; regardless of our finding that thrombotic MPO played an important role in the development of adverse LV remodeling to some extent, we failed to observe the long‐term effect of thrombotic MPO on cardiovascular death or major cardiac events. A longer follow‐up is currently on‐going, and future data could help us further clarify the true functions of MPO in thrombus.

## 6 CONCLUSIONS

Our findings strongly support that the inflammation indicated by MPO may suppress HO‐1 levels and worsen the recovery of cardiac functions in patients with AMI. We conclude that the expression of MPO in thrombi is associated with reduced HO‐1 induction and worse LV remodeling in patients with acute STEMI.

## CONFLICT OF INTEREST

None.

## Supporting information


**Figure S1** **MPO and HO‐1 are present in the thrombus collected from patients with AMI** Immunochemical staining for MPO and HO‐1 was conducted in all 41 thrombotic samples, and positive staining was detected in all samples. Representative MPO‐positive staining is shown on the left panel (labeled with arrows). Representative HO‐1‐positive staining is shown on the right panel (labeled with arrows). The magnification was 200×. AMI, acute myocardial infarction; HO‐1, heme oxygenase‐1; MPO, myeloperoxidase

## Data Availability

The data that support the findings of this study are available on request from the corresponding author. The data are not publicly available due to privacy or ethical restrictions.

## References

[1] Sianos G , Papafaklis MI , Daemen J , et al. Angiographic stent thrombosis after routine use of drug‐eluting stents in st‐segment elevation myocardial infarction: the importance of thrombus burden. J Am Coll Cardiol. 2007;50:573‐583. 17692740 10.1016/j.jacc.2007.04.059

[2] Conti CR . St‐elevation myocardial infarction: thrombus burden and prognosis. Clin Cardiol. 2008;31:3‐5. 18203114 10.1002/clc.20364 PMC6653713

[3] Brennan ML , Hazen SL . Emerging role of myeloperoxidase and oxidant stress markers in cardiovascular risk assessment. Curr Opin Lipidol. 2003;14:353‐359. 12865732 10.1097/00041433-200308000-00003

[4] Rashid I , Maghzal GJ , Chen YC , et al. Myeloperoxidase is a potential molecular imaging and therapeutic target for the identification and stabilization of high‐risk atherosclerotic plaque. Eur Heart J. 2018;39:3301‐3310. 30219874 10.1093/eurheartj/ehy419

[5] Kaya MG , Yalcin R , Okyay K , et al. Potential role of plasma myeloperoxidase level in predicting long‐term outcome of acute myocardial infarction. Tex Heart Inst J. 2012;39:500‐506. 22949765 PMC3423276

[6] Sugiyama S , Kugiyama K , Aikawa M , Nakamura S , Ogawa H , Libby P . Hypochlorous acid, a macrophage product, induces endothelial apoptosis and tissue factor expression: involvement of myeloperoxidase‐mediated oxidant in plaque erosion and thrombogenesis. Arterioscler Thromb Vasc Biol. 2004;24:1309‐1314. 15142860 10.1161/01.ATV.0000131784.50633.4f

[7] Ibanez B , James S , Agewall S , et al. 2017 esc guidelines for the management of acute myocardial infarction in patients presenting with st‐segment elevation: the task force for the management of acute myocardial infarction in patients presenting with st‐segment elevation of the european society of cardiology (esc). European Heart Journal. 2018;39:119‐177. 28886621 10.1093/eurheartj/ehx393

[8] Levine GN , Bates ER , Blankenship JC , et al. 2015 acc/aha/scai focused update on primary percutaneous coronary intervention for patients with st‐elevation myocardial infarction: an update of the 2011 accf/aha/scai guideline for percutaneous coronary intervention and the 2013 accf/aha guideline for the management of st‐elevation myocardial infarction: a report of the american college of cardiology/american heart association task force on clinical practice guidelines and the society for cardiovascular angiography and interventions. Circulation. 2016;133:1135‐1147. 26490017 10.1161/CIR.0000000000000336

[9] Teng N , Maghzal GJ , Talib J , Rashid I , Lau AK , Stocker R . The roles of myeloperoxidase in coronary artery disease and its potential implication in plaque rupture. Redox Report: Communications Free Radical Res. 2017;22:51‐73. 10.1080/13510002.2016.1256119 PMC6837458 27884085

[10] Goldmann BU , Rudolph V , Rudolph TK , et al. Neutrophil activation precedes myocardial injury in patients with acute myocardial infarction. Free Radic Biol Med. 2009;47:79‐83. 19362143 10.1016/j.freeradbiomed.2009.04.004

[11] Wainstein RV , Wainstein MV , Ribeiro JP , et al. Association between myeloperoxidase polymorphisms and its plasma levels with severity of coronary artery disease. Clin Biochem. 2010;43:57‐62. 19651119 10.1016/j.clinbiochem.2009.07.022

[12] Heslop CL , Frohlich JJ , Hill JS . Myeloperoxidase and c‐reactive protein have combined utility for long‐term prediction of cardiovascular mortality after coronary angiography. J Am Coll Cardiol. 2010;55:1102‐1109. 20223364 10.1016/j.jacc.2009.11.050

[13] Westman PC , Lipinski MJ , Luger D , et al. Inflammation as a driver of adverse left ventricular remodeling after acute myocardial infarction. J Am Coll Cardiol. 2016;67:2050‐2060. 27126533 10.1016/j.jacc.2016.01.073

[14] Reindl M , Reinstadler SJ , Tiller C , et al. Prognosis‐based definition of left ventricular remodeling after st‐elevation myocardial infarction. Eur Radiol. 2019;29:2330‐2339. 30547201 10.1007/s00330-018-5875-3 PMC6443916

[15] White HD , Norris RM , Brown MA , Brandt PW , Whitlock RM , Wild CJ . Left ventricular end‐systolic volume as the major determinant of survival after recovery from myocardial infarction. Circulation. 1987;76:44‐51. 3594774 10.1161/01.cir.76.1.44

[16] Ørn S , Ueland T , Manhenke C , et al. Increased interleukin‐1β levels are associated with left ventricular hypertrophy and remodelling following acute st segment elevation myocardial infarction treated by primary percutaneous coronary intervention. J Intern Med. 2012;272:267‐276. 22243053 10.1111/j.1365-2796.2012.02517.x

[17] Dunn LL , Midwinter RG , Ni J , Hamid HA , Parish CR , Stocker R . New insights into intracellular locations and functions of heme oxygenase‐1. Antioxid Redox Signal. 2014;20:1723‐1742. 24180287 10.1089/ars.2013.5675 PMC3961787

[18] Novo G , Cappello F , Rizzo M , et al. Hsp60 and heme oxygenase‐1 (hsp32) in acute myocardial infarction. Translational Res J Laboratory Clinic Med. 2011;157:285‐292. 10.1016/j.trsl.2011.01.003 21497776

[19] Ni J , Yang W , Shen W , Zhang R . Heme oxygenase‐1 inhibits neointimal hyperplasia in rat by histone deacetylase 2. Free Radic Res. 2018;52:1110‐1117. 30213210 10.1080/10715762.2018.1524578

[20] Posa A , Szabo R , Csonka A , et al. Endogenous estrogen‐mediated heme oxygenase regulation in experimental menopause. Oxid Med Cell Longev. 2015;2015:429713. 26064421 10.1155/2015/429713 PMC4438186

[21] Lakkisto P , Siren JM , Kyto V , et al. Heme oxygenase‐1 induction protects the heart and modulates cellular and extracellular remodelling after myocardial infarction in rats. Experimental biology and medicine Maywood NJ. 2011;236:1437‐1448. 10.1258/ebm.2011.011148 22087023

