# Supplementary figures and images for: The expression of myeloperoxidase in thrombi is associated with reduced heme oxygenase‐1 induction and worse left ventricular remodeling in patients with acute ST‐elevation myocardial infarction
Source: Clin Cardiol. 2021 Jan 6;44(3):357–63. doi: 10.1002/clc.23542 (PMC7943898; doi:10.1002/clc.23542)

Fig.S1

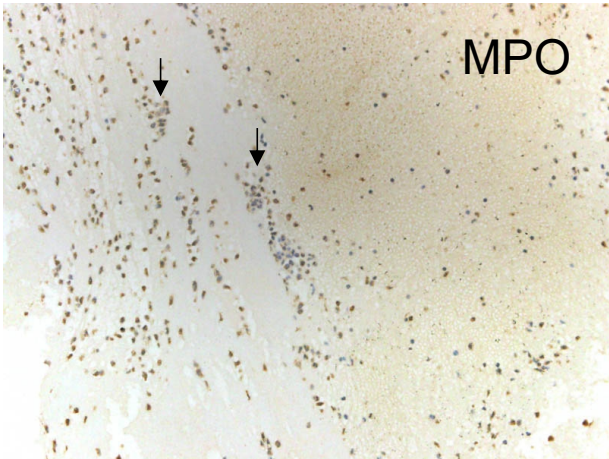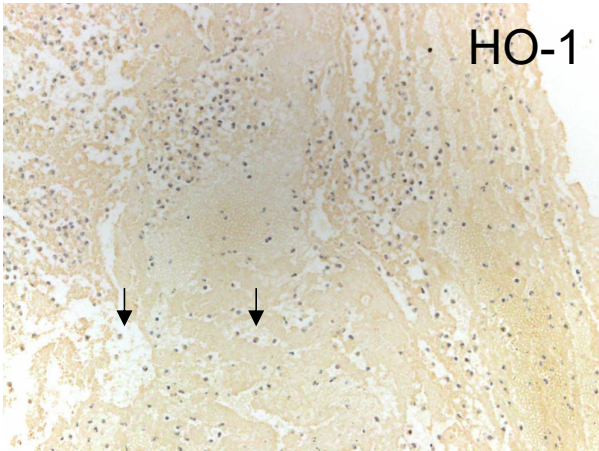

Supplement: Supplementary file 1 — Figure S1 MPO and HO‐1 are present in the thrombus collected from patients with AMI Immunochemical staining for MPO and HO‐1 was conducted in all 41 thrombotic samples, and positive staining was detected in all samples. Representative MPO‐positive staining is shown on the left panel (labeled with arrows). Representative HO‐1‐positive staining is shown on the right panel (labeled with arrows). The magnification was 200×. AMI, acute myocardial infarction; HO‐1, heme oxygenase‐1; MPO, myeloperoxidase [file CLC-44-357-s001.pdf]
